# Laser-Induced Cortical Lesions in Mice as a Model for Progressive Multiple Sclerosis Pathology

**DOI:** 10.3390/biomedicines13051195

**Published:** 2025-05-14

**Authors:** Bhavya Ojha, Bita Ramazani, Rouhin Belal, Jonathan Krieger, Maria Bloksgaard, Gabriela Teresa Lyszczarz, Dominika Rusin, Agnieszka Wlodarczyk, Una FitzGerald, Trevor Owens, Reza Khorooshi

**Affiliations:** 1 Institute of Molecular Medicine, Neurobiology Research Unit, University of Southern Denmark, DK-5230 Odense, Denmark; 2 Department of Physics, Chemistry and Pharmacy, University of Southern Denmark, DK-5230 Odense, Denmark; 3 CÚRAM, SFI Research Centre for Medical Devices, University of Galway, H91 W2TY Galway, Ireland; 4 Galway Neuroscience Centre, University of Galway, H91 TK33 Galway, Ireland

**Keywords:** progressive multiple sclerosis, cortical pathology, meningeal inflammation, demyelination

## Abstract

**Background:** The current animal models of multiple sclerosis (MS) predominantly emphasize white matter inflammation, reflecting early-stage disease. However, progressive MS (PMS) is characterized by cortical pathology, including subpial demyelination, chronic meningeal inflammation, and microglial activation, which are underrepresented in the existing models. While alternative mouse models replicate the relapsing–remitting phenotype and gray matter pathology, pathology is frequently dispersed throughout the brain, complicating the analysis of the specific lesion sites. **Methods:** To address this gap, we developed a novel model that integrates laser-induced focal demyelination with cytokine-driven meningeal inflammation to replicate the key aspects of PMS cortical pathology. **Results:** Using two-photon laser irradiation, we induced controlled subpial cortical lesions in CX3CR1-GFP mice, leading to microglial activation, astrocytosis, and focal demyelination. The addition of IFNγ-expressing adenovirus to promote meningeal inflammation which resulted in prolonged glial responses, increased immune cell infiltration, and exacerbated demyelination, mimicking the PMS-associated pathology. **Conclusions:** This model provides a powerful tool to investigate the mechanisms underlying the cortical lesion development and immune-mediated neurodegeneration in PMS. By capturing the critical aspects of cortical pathology, it enables the evaluation of therapeutic strategies targeting neuroinflammation and demyelination, ultimately aiding in the development of new treatments of progression in PMS patients.

## 1. Introduction

Multiple sclerosis (MS) is a demyelinating autoimmune disease of the central nervous system (CNS) [1], characterized by immune-mediated damage involving T cells, B cells, and macrophages/microglia [2]. MS pathology manifests through demyelinated lesions in both white and gray matter, with variable inflammation, axonal damage, and gliosis, depending on the disease stage [2]. MS progresses as a continuum, and subpial cortical lesions are a hallmark of the progressive forms of MS (PMS) [3]. These lesions are associated with the heightened activation of microglia/macrophages and meningeal inflammation, with meningeal T and B cell involvement correlating with the extent of cortical demyelination [4,5,6]. Increasing evidence suggests that distinct immune signaling pathways in immune cells may drive the early autoimmune responses in MS [7,8]. These pathways may serve not only as markers of early disease activity but also as potential targets for selective therapeutic intervention before irreversible CNS damage occurs. Understanding how such mechanisms evolve across the disease stages underscores the need for model systems that can accurately replicate both the early and progressive features of MS.

Animal models have significantly advanced our understanding of the pathophysiology of multiple sclerosis (MS). Traditional models, such as experimental autoimmune encephalomyelitis (EAE), have predominantly concentrated on the inflammation in and demyelination of white matter, which are characteristic of the early stages of MS. Alternative mouse models that employ varied immunization strategies successfully replicate the relapsing–remitting phenotype of MS and incorporate gray matter pathology, thereby providing a more comprehensive representation of the disease in its later stages [9,10,11,12]. Add-on strategies such as lentivirus-induced cytokine overexpression promote chronic inflammation and demyelination [13]. However, the pathology in such models is often dispersed throughout the brain, complicating the analysis of the specific lesion sites. To overcome this limitation, we are developing models that induce spatially defined focal cortical lesions, enabling the precise examination of MS mechanisms and facilitating targeted therapeutic strategies.

Laser technology offers various biomedical applications, including in vivo imaging through two-photon excitation microscopy, which minimizes phototoxicity and improves the imaging depth [4,14]. This minimally invasive approach has also been used to modulate the blood–brain barrier (BBB) and deliver targeted molecules to the brain cortex [4]. Low-level laser therapy has shown therapeutic potential in conditions such as osteoarthritis, neuropathic pain, depression, and ulcers [15,16]. In murine models, it has demonstrated benefits for demyelinating diseases [17,18], and its focal nature allows for precise tissue ablation [19].

We here report the induction of subpial cortical lesions using minimally invasive two-photon laser irradiation. The laser induces a focal injury by converting absorbed light energy into heat, creating localized damage. This report describes the characterization of PMS-like cortical pathology following a laser-induced injury, including subpial cortical gliosis and demyelination and immune cell infiltration, and the impact of meningeal inflammation on these. It is anticipated that this approach will allow the testing of potential therapeutic interventions to target the unique features of cortical pathology, with the goal of slowing or halting the disease progression in PMS patients.

## 2. Results

### 2.1. Glial Activation and Immune Response After a Laser-Induced Cortical Injury

Histopathological analysis using hematoxylin and eosin (H&E) staining was conducted to assess the effects of a laser-induced cortical injury in mice. The laser was focused to a depth of 500 µm within the cortex, targeting a specific region to induce an injury. Brains were collected at 3 and 5 days post-irradiation (DPI) for further analysis.

**Figure 1:**
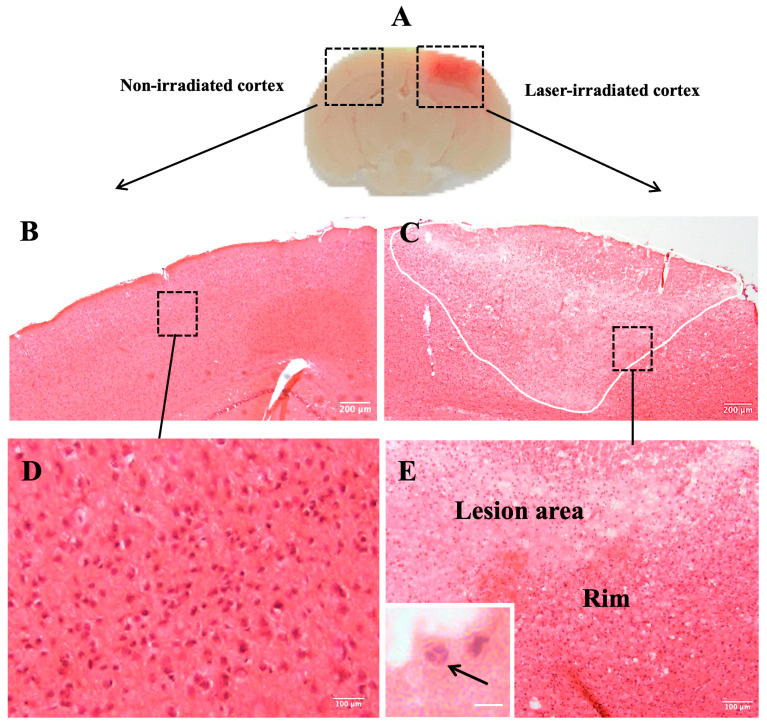
Histopathological analysis of brain tissue. (**A**) Laser-induced bleeding in the irradiated cortex is visible in isolated brains as well as in cryostat sections at 3 DPI. (**B**) Hematoxylin and eosin (H&E) staining of non-irradiated cortex of mouse brain showing no histological changes. (**D**) Micrograph shows higher magnification of inset box in (**B**). (**C**) Histological examination using H&E staining confirms loss of tissue integrity and increased cellularity in the irradiated cortex (white outline indicates lesion area). Box inset indicates lesion rim, visible in higher magnification in (**E**), showing cellular aggregates in the lesion rim. Inset in (**E**) shows cellular aggregates in laser-irradiated cortex (arrows indicate possible PMNs). (n = 9). Scale bar: 200 μm (**B**,**C**), 100 µm (**D**,**E**), 10 µm (inset).

At 3 DPI, laser-induced bleeding was evident in both isolated brains and cryostat sections of the irradiated cortex (Figure 1A). This bleeding, directly resulting from the laser injury, confirmed tissue disruption. Histological examination at this time point revealed significant damage in the irradiated cortex, including the loss of tissue integrity and increased cellularity (Figure 1C). In contrast, H&E staining of the non-irradiated cortex showed no histological alterations, confirming that the injury was confined to the irradiated region and was not a consequence of skull thinning (Figure 1B). High-magnification analysis of the non-irradiated cortex revealed no abnormalities, further supporting the localized nature of the injury (Figure 1D). The lesion area in the irradiated cortex, shown in Figure 1C, is delineated by a white outline, with the lesion rim visible in the inset, where cell aggregation is clearly observed. At higher magnification, cellular aggregates within the lesion rim revealed a variety of cells, including those exhibiting polymorphonuclear (PMN) morphology. These PMN cells, characterized by multi-lobed nuclei, are indicative of infiltrating immune cells, such as neutrophils (Figure 1E, inset).

**Figure 2:**
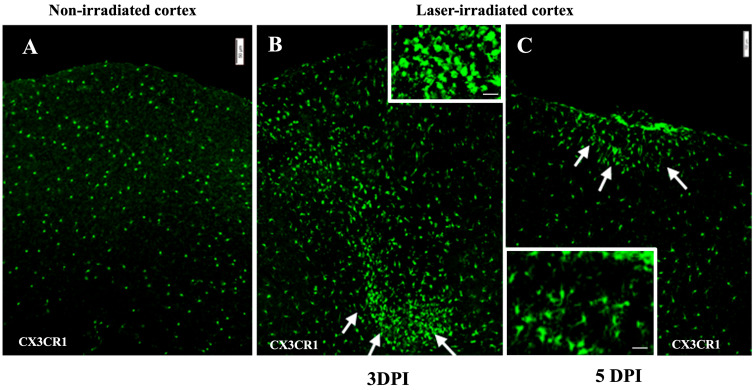
Microglia in laser-irradiated mice: the micrographs show green microglia in CX3CR1^GFP/+^ mice. (**A**) Microglia in the unirradiated cortex appear unaffected by skull thinning. (**B**) Microglia cluster around lesions in the irradiated cortex at 3 DPI. The microglia form aggregates, indicated by white arrows. Inset shows higher magnification. (**C**) Microglia in irradiated cortex at 5 DPI. The cluster of microglia is more compact and localized to a smaller area indicated by white arrows. Inset: microglia within clusters show amoeboid morphology. The experiment was independently performed twice (n = 12). Scale bar, 50 µm, 10 µm (inset).

Microglial activation was assessed using GFP fluorescence in CX3CR1-GFP mice, which allows for the visualization of microglial cells. At 3 DPI, microglial clusters were particularly prominent around the periphery of the lesion (Figure 2). The microglia exhibited an amoeboid morphology, characterized by reduced processes, indicative of an activated and phagocytic state (Figure 2B inset). At 5 DPI, microglial aggregates were still present but reduced and were confined to the subpial regions of the cortex (Figure 2C).

**Figure 3:**
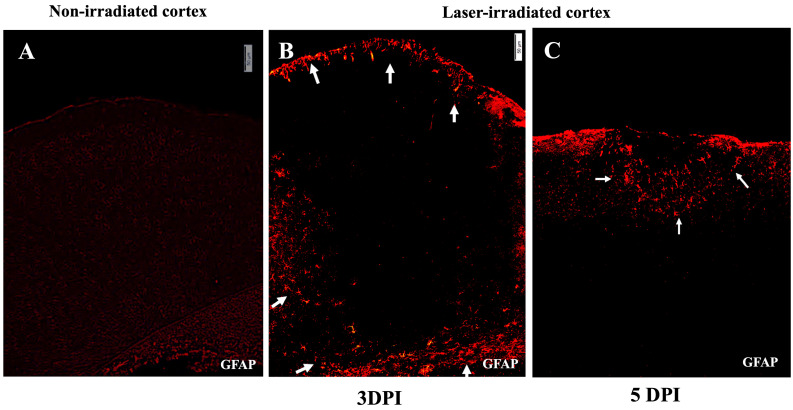
Astrogliosis after irradiation. (**A**) Micrograph of non-irradiated cortex showing normal low-level GFAP staining. (**B**,**C**) Astrocytic activity in irradiated cortex indicated by GFAP-stained astrocytes in red at 3 DPI (**B**) and 5 DPI (**C**). (**B**) GFAP reactive astrocytes align around the lesion region and at the glia limitans, localizing at the periphery of the lesion, indicated by white arrows. (**C**) At 5 DPI, the region of astrocytic activity has condensed, with a reduced area of gliosis proximal to the subpial region, indicated by white arrows. Activated astrocytes were not observed deeper in the cortex. The experiment was independently performed twice (n = 12). Scale bar, 50 µm.

Astrocytic activation was assessed using GFAP staining, which labels reactive astrocytes. At 3 DPI, GFAP-stained astrocytes with a reactive morphology were found to align with the microglial aggregates at the lesion periphery (Figure 3). This alignment between activated microglia and astrocytes suggests a coordinated glial response to the injury. In the non-irradiated cortex, no aggregation or activation-associated morphological changes were observed in either microglia or astrocytes (Figure 2A and Figure 3A). At 5 DPI, astrocytic aggregates were still detectable but, like the microglia, were reduced and confined to the subpial regions of the cortex (Figure 3C).

**Figure 4:**
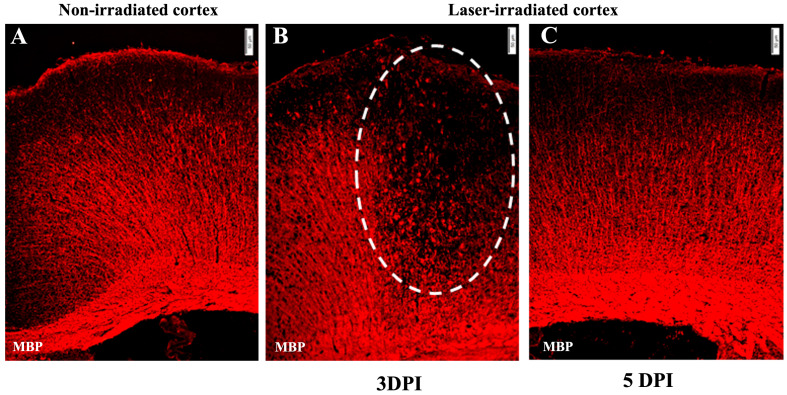
Demyelination: micrographs show anti-MBP staining (red). (**A**) Undisrupted myelin in the non-irradiated cortex. (**B**) Demyelination at 3 DPI in the irradiated cortex. MBP staining shows disrupted myelin, with myelin debris in the lesion, indicated by the dashed line circle. (**C**) MBP staining at 5 DPI indicates no loss of myelin. The experiment was independently performed twice (n = 12). Scale bar, 50 µm.

Demyelination was evident at 3 DPI, as indicated by a significant loss of myelin basic protein (MBP) staining in the center of the lesion (Figure 4). However, by 5 DPI, demyelination was no longer evident (Figure 4C).

**Figure 5:**
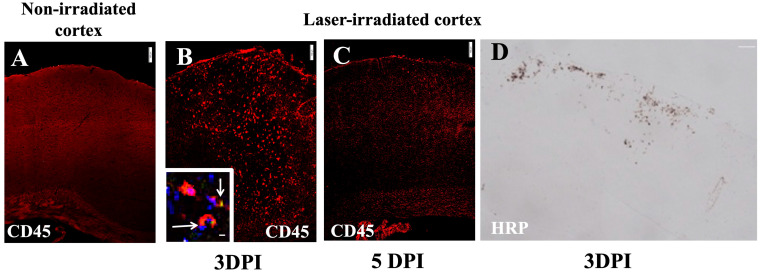
Leukocyte infiltration (red) in cortex after irradiation: (**A**) CD45 staining in non-irradiated cortex showing no infiltration. (**B**,**C**) Intense infiltration at 3 DPI, lessening at 5 DPI (**C**). Inset in (**B**) shows GR1 + (green) and CD45+ (red) colocalization (arrow), DAPI (nuclei, blue). Scale bar, 100 µm. (**D**) BBB disruption in irradiated cortex at 3 DPI: micrograph shows HRP that leaked from peripheral blood following IV injection 15 min earlier. The experiment was independently performed twice (n = 12). Scale bar, 200 µm. 10 µm (inset).

Leukocyte infiltration into the irradiated cortex was assessed using CD45 staining, which identifies blood-derived immune cells. At 3 DPI, there was substantial infiltration of CD45-positive cells into the lesion site (Figure 5B). These infiltrating immune cells included granulocytes, as indicated via Gr1 staining (Figure 5B inset). The infiltration at 5DPI as indicated via CD45 staining was restricted more towards the subpial region which also corresponded to the microglial and astrocytic activity (Figure 5C). The micrograph indicates the resolution of infiltration to a large extent at 5 DPI (Figure 5C). Furthermore, the detection of horseradish peroxidase (HRP) tracer revealed a blood–brain barrier (BBB) breakdown in the same regions of the irradiated cortex, indicating that the laser injury resulted in localized BBB dysfunction (Figure 5D).

### 2.2. Meningeal Inflammation Prolongs Glial and Immune Responses to a Cortical Injury

To investigate the impact of meningeal inflammation on the glial and immune responses following a cortical injury, CX3CR1-GFP transgenic mice were administered an interferon-gamma (IFNγ)-expressing adenovirus via intrathecal injection one week before laser irradiation. The administration of IFNγ significantly influenced the glial and immune responses following cortical injury and prolonged the activation of microglia and astrocytes. We have previously shown that intrathecal IFNγ-expressing adenovirus did not itself lead to inflammation [20].

**Figure 6:**
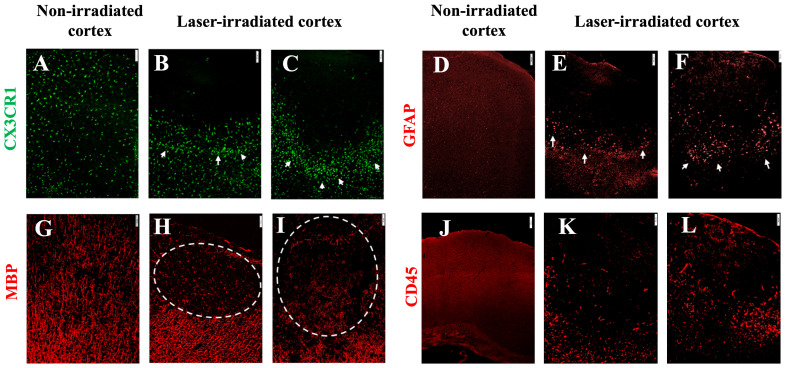
Gliosis and infiltration in irradiated mice with meningeal inflammation (intrathecal Ad-IFN-γ 1 week before irradiation). (**A**–**C**) Microglia in CX3CR1^gfp/+^ mice. (**A**) Non-irradiated cortex. (**B**) At 3 DPI, microglia aggregated at the periphery of the lesion. (**C**) The size of the cluster and the density remained extensive at 5 DPI. (**D**–**F**) Astrocytes stained with anti-GFAP. (**D**) Non-irradiated cortex. (**E**,**F**) Activated astrocytes at the periphery of the lesion (white arrows) at 3 DPI (**E**) and 5 DPI (**F**), aligning with microglia in (**B**,**C**). (**G**) MBP staining in non-irradiated cortex. (**H**,**I**) Myelin disruption at 3 DPI (**H**) and 5DPI (**I**), indicated by the dashed line circles. (**J**–**L**) Leukocyte infiltration (CD45 staining). (**J**) Non-irradiated cortex. (**K**,**L**) Intense infiltration at 3 DPI (**K**) and 5DPI (**L**). The experiment was independently performed twice (n = 16), scale bar, 50 µm.

At both 3 and 5 DPI, IFNγ-treated mice displayed more dense and hypercellular glial aggregates, prominent around the periphery of the lesion. This amplification of the glial response was particularly evident for microglia (Figure 6B,C) and astrocytes (Figure 6E,F). The increased density and aggregation of microglia and astrocytes were more pronounced within the lesion core and surrounding regions. Notably, no significant microglial or astrocytic activation was observed in the non-irradiated cortex (Figure 6A,D), confirming that the glial response was injury specific and modulated by IFNγ.

Demyelination, as evidenced by a loss of MBP staining, was pronounced in the lesion core at both 3 and 5 DPI in IFNγ-treated animals compared to non-irradiated cortex (Figure 6H,I).

The immune response to a cortical injury was further evaluated via CD45 staining. At 3 DPI, IFNγ-treated animals displayed a significantly increased number of infiltrating leukocytes in the irradiated cortex (Figure 6J). This enhanced the immune cell infiltration and persisted at 5 DPI (Figure 6K). Compared to the non-irradiated cortex (Figure 6J), leukocyte infiltration was evident in the IFNγ-treated lesion, indicating that meningeal inflammation likely contributes to the recruitment and persistence of immune cells at the site of an injury.

A comparative summary of the pathological features in the laser only and laser + IFNγ groups is presented in Supplementary Table S1. The table highlights the key differences in glial activation, demyelination, immune cell infiltration, and BBB disruption, illustrating the exacerbated neuroinflammatory response elicited by IFNγ in the context of a laser-induced injury.

**Figure 7:**
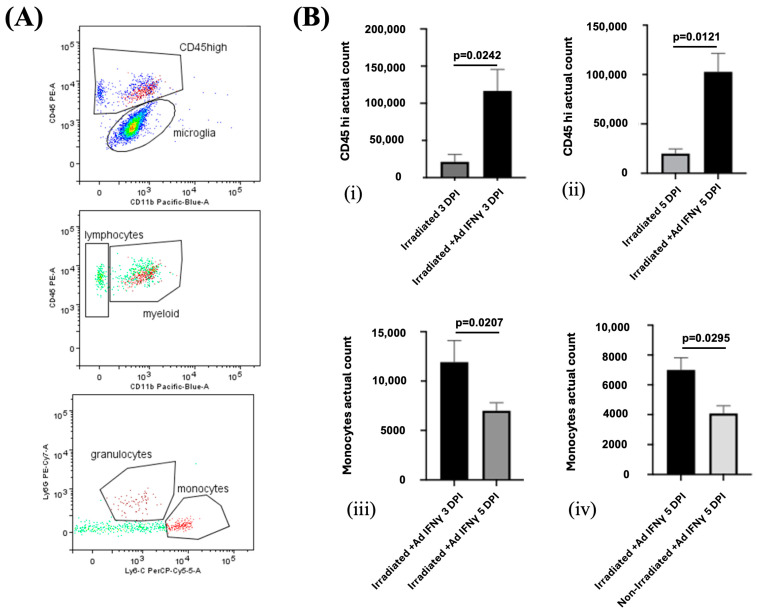
(**A**) Representative flow cytometry profiles from individual ipsilateral hemisphere suspension from irradiated mice. Flow cytometry gating strategy to distinguish CD45^high^ leukocytes from Cd45^dim^ microglia. Monocytes (CD45^high^CD11b^high^Ly6C^high^) and granulocytes (CD45^high^CD11b^high^Ly6G^high^) were gated from CD45high. (**B**) Infiltration of leukocytes is enhanced by meningeal IFNγ in laser irradiated group. The total number of infiltrating CD45^high^ leukocytes is enhanced at both 3 DPI ((**i**), *p* = 0.0242) and 5 DPI ((**ii**), *p* = 0.0121). (**iii**) The total number of Ly6chighcells, representing monocytes, in the irradiated cortex was lower at 5 DPI than at 3 DPI (*p* = 0.0207). (**iv**) Significantly more Ly6c^high^ monocytes infiltrated the irradiated than the non-irradiated cortex (*p* = 0.0295). The experiment was independently performed twice (n = 4).

Flow cytometry further confirmed the presence of a higher number of CD45^high^ cells, particularly monocytes, in the irradiated cortex of IFNγ-treated mice compared to the controls (Figure 7). The number of monocytes was increased at both 3 and 5 DPI, suggesting that IFNγ not only promotes the initial infiltration of immune cells but also sustains their presence in the lesion site over time. Although some monocytes were detected in the non-irradiated cortex at 5 DPI, their numbers were about half those observed in the irradiated side, reinforcing the injury-specific nature of the immune response (Figure 7).

## 3. Discussion

Our study presents a focal cortical pathology model in mice that recapitulates the key features of cortical inflammation, meningeal cytokine-driven inflammation, and cortical demyelination, all of which are characteristics of neuroinflammatory diseases such as MS. Using high-wavelength laser irradiation, a minimally invasive approach, we induced targeted brain lesions, allowing us to monitor the temporal dynamics of the inflammatory response with high precision through two-photon laser microscopy. This method facilitates the real-time analysis of the cellular and molecular processes following a cortical injury.

In this model, we employed CX3CR1GFP/+ mice, which express green fluorescent protein (GFP) under the control of the CX3CR1 promoter, enabling the in vivo tracking of the microglial behavior in both their resting and activated states. Following a cortical injury, microglia exhibited a rapid and robust aggregation around the injury site, demarcating a clear transition from resting to activated states. Activated microglia assumed an amoeboid morphology, characteristic of a phagocytic phenotype, consistent with a response aimed at clearing cellular debris, including myelin fragments. In the context of neuroinflammatory diseases such as MS, the phagocytic activity of microglia is crucial for the removal of myelin debris. However, in MS, the efficiency of this process is often compromised, leading to the accumulation of myelin fragments that hinder remyelination and exacerbate neurodegenerative pathology [21].

Our results are in agreement with a previous study, which showed that microglial activation occurs rapidly after a cortical injury and persists for several days [19]. By 5 days post-irradiation, we observed a reduction in the size of microglial aggregates, suggesting partial resolution of the injury. Astrocytes, another critical cell type involved in glial responses, also became reactive, forming dense aggregates around the injury site. This astrocytic response is typical of glial scar formation, which can protect the surrounding tissue but may physically hinder remyelination and repair [22]. The formation of the glial scar may thus act as a double-edged sword, promoting tissue repair while simultaneously obstructing the regenerative processes that are necessary for functional recovery.

To examine the role of persistent meningeal inflammation in modulating these responses, we introduced IFNγ into the system using an adenoviral vector [20]. This has been previously validated to induce bioactive IFNγ in the cerebrospinal fluid of the treated animals, without the induction of inflammation in the absence of other stimuli [20]. IFNγ is a cytokine commonly elevated in the cerebrospinal fluid of MS patients and is known to exacerbate the inflammatory responses [5]. In our model, IFN-γ supplementation resulted in enhanced and prolonged microglial and astrocytic activation, as well as more pronounced and persistent demyelination at 5 DPI. These findings suggest that meningeal inflammation plays a crucial role in prolonging the inflammatory response and exacerbating tissue damage [23]. Notably, astrocytic infiltration into the lesion was significant by 3 DPI, potentially contributing to the formation of the glial scar, which, while aiding in tissue repair, may also limit remyelination [22].

Further investigation revealed an increased presence of CD45-positive immune cells, particularly monocytes, in the IFNγ-treated animals. Flow cytometry confirmed a significant elevation in the number of CD45^high^ cells, specifically monocytes, in the irradiated cortex of IFNγ-treated mice compared to the controls. These results support the hypothesis that meningeal inflammation synergizes with a laser-induced injury to enhance the immune cell infiltration into the lesion site. This enhanced immune cell recruitment mirrors the observations in MS, where glial activation and immune cell infiltration are the major contributors to lesion formation and disease progression [7,24,25,26].

We also observed blood–brain barrier (BBB) disruption in all the laser-irradiated conditions, consistent with previous reports showing transient BBB permeability following an injury [4]. However, the prolonged BBB disruption observed in our model, likely due to the high-wavelength, long-duration irradiation, suggests more sustained BBB breakdown. This persistent disruption may facilitate the ongoing immune cell infiltration, and sustained inflammatory response as observed in progressive neurodegenerative diseases such as MS [11,27]. These findings highlight the potential role of BBB dysfunction in maintaining chronic inflammation and immune activation, which are hallmarks of progressive MS pathology [4].

Bleeding in irradiated subpial cortex and the resolution of lesions by 5 DPI in the absence of IFNγ-expressing adenovirus can indicate an acute cortical injury. Although a cerebral injury is not considered to initiate MS lesions, we have exploited this mild laser-induced trauma to model chronic demyelinating PMS-like lesions in mice. Importantly, despite the fact that skull thinning likely also involves mild trauma, there was no evidence for gliosis or tissue disruption in the unirradiated cortex, emphasizing the key role for laser irradiation to play. The immediate microglial response to cerebral trauma which is described as a priority for restoring a stable microenvironment [19] is potentiated by meningeal IFNγ, leading to chronic demyelination. Thus, in conjunction with prior immune reactivity such as would occur in RRMS, laser injury-induced events develop towards a PMS-like cortical pathology. This complements the existing animal models for MS- and PMS-like pathology, in particular potentially offering insight into non-immune-initiated, CNS-endogenous cellular and molecular processes. Unlike EAE with cytokine overexpression models, which primarily reflect immune-driven, white matter inflammation, and diffuse pathology throughout the brain, our model allows the focused study of cortical gray matter injury. The addition of IFN-γ introduces an inflammatory component helping bridge the immune and intrinsic CNS mechanisms in PMS-related pathology [28].

While our model provides valuable insights into the dynamics of glial activation, immune cell infiltration, and de- and remyelination in cortical injury and inflammation, it is important to acknowledge its limitations. The laser-induced lesions in our model, while replicating the key features of the cortical pathology observed in MS, differ from the spontaneous lesions seen in human MS, particularly in terms of their location and formation mechanism. In MS, cortical lesions are classified as leukocortical, intracortical, subpial, and type IV, which is a combination of subpial and intracortical lesions. These lesions are associated with inflammation and are considered a major contributor to disease progression [29]. Additionally, while we observed sustained BBB disruption, inflammation, and demyelination, the degree of these responses may not fully recapitulate the chronic, progressive inflammation characteristic of the progressive forms of MS [9]. Our study focuses on short-term outcomes (3 and 5 DPI). Although beyond the current scope, longer-term evaluations would be important to determine whether chronic PMS-like pathology develops. Nonetheless, this model offers a valuable platform for investigating the cellular and molecular mechanisms underlying cortical inflammation, demyelination, and glial activation, and for testing the potential therapeutic interventions aimed at remyelination and immune modulation, in the broadest context possible. Future research directions may also be highlighted.

## 4. Materials and Methods

### 4.1. Mice

C57/BL6J female mice were purchased from Taconic Europe A/S (Lille Skensved, Denmark). CX3CR1^GFP/GFP^ mice on a C57/BL6J background were purchased from Jackson Laboratory and crossed with C57/BL6J mice to obtain heterozygous colonies of mice that were bred and maintained in the Biomedical Laboratory, University of Southern Denmark (Odense, Denmark). Heterozygous CX3CR1^GFP/+^ reporter mice were used to visualize microglia.

All the experiments were approved by the Danish Animal Experiments Inspectorate (approval number: 2020-15-0201-00652).

### 4.2. Experimental Setup

Unilateral two-photon laser irradiation was applied to create a focal cortical lesion approximately 500 µm deep in the subpial cortex. Mice were sacrificed at two time points, 3 days post-irradiation (DPI) and 5 DPI, to assess both the early and intermediate glial and immune responses to an injury. Brains were subsequently processed for immunohistochemistry and flow cytometry to evaluate the glial activation, myelin integrity, and immune cell infiltration.

A separate cohort of mice received intrathecal Ad-IFNγ one week prior to irradiation to assess the effects of heightened inflammatory signaling on the injury response. The intrathecal administration of Ad-IFNγ was used to model the effects of chronic inflammation, known to exacerbate the neurodegenerative and injury responses in the brain.

To ensure an accurate comparison between the irradiated and non-irradiated hemispheres, bilateral skull thinning was performed, with only one hemisphere receiving laser irradiation, allowing the non-irradiated hemisphere to serve as a control. This design enabled the precise assessment of the localized effects of a laser-induced injury and IFN-γ treatment, independent of surgical or experimental artifacts such as skull thinning.

### 4.3. Laser Irradiation

Female mice aged 10–12 weeks old were anesthetized using a subcutaneous injection of a mixture of midazolam (0.1 mg/g) and hypnorm (0.1 mg/g) in sterile water.

The mice received 0.1 mg/kg Temgesic (RB Pharmaceuticals Limited, Berkshire, UK) thrice at an interval of 6–8 h as an analgesic and isotonic water to prevent dehydration subcutaneously. The head of the mouse was fixed in a stereotactic frame (Kopf Instruments, Tujunga, CA, USA), and a midline skin incision was made to expose the skull. The skull was thinned on both sides behind the bregma (1 mm^2^ and about 30 µm deep). The mouse was then transferred to the microscope stage, where the head of the mouse was fixed in a stereotactic device (SDU workshop, Odense, Denmark). The microscope was an upright Olympus FV1000 MPE microscope equipped with an Olympus LCPlanFI 40×/0.60 NA objective (Olympus, Bartlett, TN, USA) and a MaiTai^®^ DeepSee^TM^ (Spectra Physics, Mölnlycke, Sweden) MPE laser. The laser wavelength was set to a 760 nm excitation and the maximum power output. The laser injury was introduced in the right hemisphere of the thinned skull by scanning a region of 500 µm × 800 µm × 800 µm with a step size of 50 µm/slice and 20 µs/pixel dwell time. The laser irradiation experiments were performed at the Danish Molecular Biomedical Imaging Center (DaMBIC, University of Southern Denmark, Odense, Denmark).

An intrathecal injection of adenovirus encoding murine interferon gamma (IFN-γ) was performed.

The mice were anesthetized via the inhalation of isoflurane (Abbott Laboratories, Abbott Park, IL, USA). A 30-gauge needle (bent 55° with a 2 mm tip) was attached to a 50 μL Hamilton syringe to perform an intrathecal injection into the cisterna magna. The mice received 10^7^ pfu of a replication-defective type 5 E1 E3 deleted adenovirus encoding murine IFN-γ (Ad-IFNγ), driven by a Cytomegalovirus immediate early promoter [20]. Ten microliters of Ad-IFNγ in sterile Phosphate Buffered saline (PBS) (10^9^ pfu/mL) was injected over 30 s. Supplementary Figure S1 provides a schematic representation of the study design.

### 4.4. Tissue Processing

The mice were euthanized using an overdose of sodium pentobarbital (100 mg/kg, Glostrup Apotek, Glostrup, Denmark) injected intraperitoneally, and perfused transcardially with ice-cold PBS before brain isolation.

For histology, the brains were post-fixed with 4% paraformaldehyde (PFA) for a minimum of 1 h, immersed in 30% sucrose in PBS for a week at 4 °C, then frozen in cryostat embedding medium (Killik, Milano, Italy) via immersion in 2-methylbutane (Sigma-Aldrich, Søborg, Denmark) in liquid nitrogen. With a 14 μm thickness, tissue sections of frozen brains were cut on a cryostat (Leica, Copenhagen, Denmark). These sections were then mounted on Superfrost^TM^ Plus Adhesion Microscope Slides (Epredia Netherlands B.V., Breda, The Netherlands).

To evaluate the BBB integrity, the mice received 2 mg of horseradish peroxidase (HRP type VI-A Sigma-Aldrich P6782) in 100 μL of PBS via intravenous injection. The mice were transcardially perfused 15 min later, following which the brain was isolated and processed for histology. The sections were developed with 3, 3′-diaminobenzidine.

### 4.5. Flow Cytometry

For flow cytometry, the brains were isolated after perfusing the mice transcardially, then placed in ice-cold PBS for the further procedure to take place.

The irradiated (ipsilateral) and non-irradiated (contralateral) regions were separately isolated, and single-cell suspensions were generated by forcing the tissue through a 70 μm cell strainer (Falcon, Corning, NY, USA) with Hank’s buffered salt solution (HBSS, Gibco, Waltham, MA, USA) supplemented with 2% fetal bovine serum (FBS, Merck, Darmstadt, Germany). Myelin was removed from the cell suspension after centrifugation on 37% Percoll (GE Healthcare Cytvia, Biosciences, Uppsala, Sweden) in a buffer consisting of 45 mL of 10× PBS, 3 mL of HCI, 132 mL of water, and a pH of 7.2. The cells were then incubated with BD Truecount cell counting beads followed by blocking buffer containing HBSS, 2% FBS, anti CD16/32 antibody (1 µg/mL, Clone 2.4G2, BD Biosciences, San Jose, CA, USA), and Syrian hamster IgG (50 μg/mL, Jackson Immuno Research Laboratories Inc., West Grove, PA, USA). The cells were then labeled using fluorophore-conjugated antibodies: anti-CD45 (Biolegend, 30-F11), anti-CD11b (Biolegend, M1/70), anti-Ly6C (Biolegend, HK1.4), and anti-Ly6G (BD, 1a8).

The cell viability was checked by staining the cells with LIVE/DEAD™ Fixable Green Dead Cell Stain Kit with a 488 nm excitation. Data were collected on an LSRII™ flow cytometer (BD Biosciences, San Jose, CA, USA) and analyzed using FlowLogic software version 8.7 (Invai Technologies, Victoria, Australia).

### 4.6. Histology

For the fluorescent staining, sections were incubated with primary antibodies followed by secondary antibodies. The nuclei were visualized via DAPI staining. Sections were mounted using DAKO fluorescent mounting medium. Myelin was stained using a rat anti-myelin basic protein (MBP) antibody (Abcam, Cambridge, UK), followed by incubation using donkey-anti rat IgG (Thermo Fisher, Waltham, MA, USA). Astrocytes were identified via staining with an anti-glial fibrillary acidic protein (GFAP) antibody conjugated with CY3 (Merck, Darmstadt, Germany). The immune cell infiltration was investigated using an AlexaFluor 594-conjugated anti-CD45 antibody (Biolegend, San Diego, CA, USA).

Photomicrographs were taken using an Olympus DP80 digital camera mounted on an Olympus BX63 microscope and processed using Fiji.

### 4.7. Statistical Analysis

The Rout test (Q = 1) was used to eliminate outliers before the statistical testing. The data were analyzed by using the non-parametric Student’s *t*-test followed by the Mann–Whitney test. Statistical analyses were performed using GraphPad Prism version 9 (GraphPad Software, San Diego, CA, USA). The results are presented as means ± SEM. *p* values < 0.05 were considered statistically significant.

## Data Availability

The data will be available upon request.

## Supplementary Materials

The following supporting information can be downloaded at: https://www.mdpi.com/article/10.3390/biomedicines13051195/s1, Figure S1: Experimental setup: (a) Mice were organized in four groups. The groups were segregated as follows-. Group 1: CX3CR1GFP/+ mice were intrathecally injected with Ad-IFNγ 7 days prior to the experiment, irradiated on Day 0 and sacrificed 3DPI. Group 2: CX3CR1GFP/+ mice were irradiated on Day 0 and sacrificed 3DPI. Group 3: CX3CR1GFP/+ mice were intrathecally injected with Ad-IFNγ 7 days prior to the experiment, irradiated on Day 0 and sacrificed 5DPI. Group 4: CX3CR1GFP/+ mice were irradiated on Day 0 and sacrificed 5DPI. (b) The skull was thinned prior to irradiation. The area of thinned skull in both hemispheres is marked by black stippled circles. The right hemisphere was irradiated while the left (contralateral) was not. The contralateral region also served as negative control. (c) Representative model of irradiation targeting in brain; Table S1: Comparative analysis of irradiated and meningeal IFN-γ + irradiation mouse models. Key features across four experimental subgroups are presented below. Feature intensity is graded as follows: Strong (++), Moderate (+), or Absent (−).
