# Supplementary material for: Laser-Induced Cortical Lesions in Mice as a Model for Progressive Multiple Sclerosis Pathology
Source: Biomedicines. 2025 May 14;13(5):1195. doi: 10.3390/biomedicines13051195 (PMC12109324; doi:10.3390/biomedicines13051195)
Supplement: Supplementary file 1 [file biomedicines-13-01195-s001.zip › biomedicines-3611428-supplementary.pdf]

biomedicines

MDPI

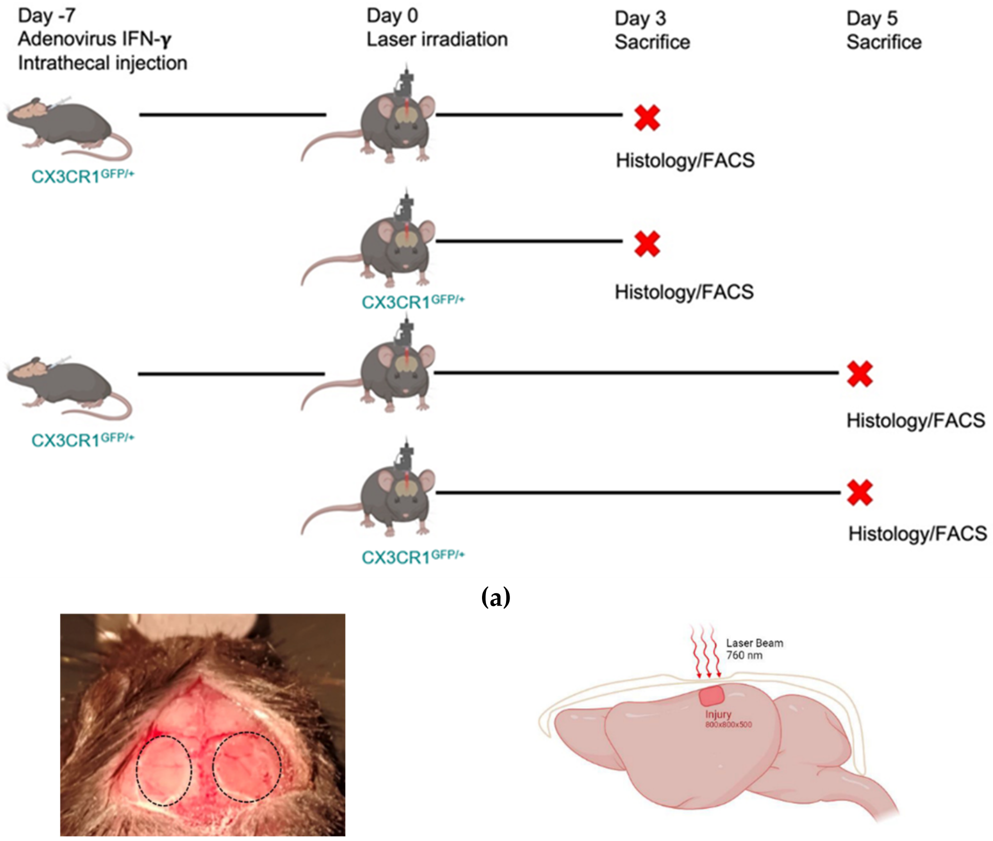

(a)

(b) (c)

Figure S1. Experimental setup: a) Mice were organized in four groups. The groups were segregated as follows-. Group 1: CX3CR1GFP/+ mice were intrathecally injected with Ad-IFNγ 7 days prior to the experiment, irradiated on Day 0 and sacrificed 3DPI. Group 2: CX3CR1GFP/+ mice were irradiated on Day 0 and sacrificed 3DPI. Group 3: CX3CR1GFP/+ mice were intrathecally injected with Ad-IFNγ 7 days prior to the experiment, irradiated on Day 0 and sacrificed 5DPI. Group 4: CX3CR1GFP/+ mice were irradiated on Day 0 and sacrificed 5DPI. b) The skull was thinned prior to irradiation. The area of thinned skull in both hemispheres is marked by black stippled circles. The right hemisphere was irradiated while the left (contralateral) was not. The contralateral region also served as negative control. c) Representative model of irradiation targeting in brain.

Table S1. Comparative analysis of irradiated and meningeal IFN-γ + irradiation mouse models. Key features across four experimental subgroups are presented below. Feature intensity is graded as follows: Strong (++), Moderate (+), or Absent (–).

	**Laser only**		**Laser + IFN-γ**	
	**3 DPI**	**5 DPI**	**3 DPI**	**5 DPI**
- Lesion size	++	+	++	++
-Microglial activation<br>-Localization	++<br>in and around the lesion	+<br>subpial cortex	++<br>in the lesion periphery	++<br>in the lesion periphery
-Astrocytic activation<br>-Localization	++<br>around lesion	+<br>subpial cortex	++<br>in and around lesion	++<br>in and around lesion
Demyelination	++	-	++	++
-CD45 Infiltration<br>-Localization	+<br>Inside the lesion	+<br>Inside the lesion	++<br>Inside the lesion	++<br>Inside the lesion
BBB integrity	Disrupted	Disrupted	Disrupted	Disrupted
